# Improved quantification of tumor adhesion in meningiomas using MR elastography-based slip interface imaging

**DOI:** 10.1371/journal.pone.0305247

**Published:** 2024-06-25

**Authors:** Keni Zheng, Matthew C. Murphy, Emanuele Camerucci, Aaron R. Plitt, Xiang Shan, Yi Sui, Armando Manduca, Jamie J. Van Gompel, Richard L. Ehman, John Huston, Ziying Yin

**Affiliations:** 1 Radiology, Mayo Clinic, Rochester, MN, United States of America; 2 Neurology, Mayo Clinic, Rochester, MN, United States of America; 3 Neurosurgery, Mayo Clinic, Rochester, MN, United States of America; 4 Physiology and Biomedical Engineering, Mayo Clinic, Rochester, MN, United States of America; 5 Otolaryngology, Mayo Clinic, Rochester, MN, United States of America; Union Hospital, Tongji Medical College, Huazhong University of Science and Technology, CHINA

## Abstract

Meningiomas, the most prevalent primary benign intracranial tumors, often exhibit complicated levels of adhesion to adjacent normal tissues, significantly influencing resection and causing postoperative complications. Surgery remains the primary therapeutic approach, and when combined with adjuvant radiotherapy, it effectively controls residual tumors and reduces tumor recurrence when complete removal may cause a neurologic deficit. Previous studies have indicated that slip interface imaging (SII) techniques based on MR elastography (MRE) have promise as a method for sensitively determining the presence of tumor-brain adhesion. In this study, we developed and tested an improved algorithm for assessing tumor-brain adhesion, based on recognition of patterns in MRE-derived normalized octahedral shear strain (NOSS) images. The primary goal was to quantify the tumor interfaces at higher risk for adhesion, offering a precise and objective method to assess meningioma adhesions in 52 meningioma patients. We also investigated the predictive value of MRE-assessed tumor adhesion in meningioma recurrence. Our findings highlight the effectiveness of the improved SII technique in distinguishing the adhesion degrees, particularly complete adhesion. Statistical analysis revealed significant differences in adhesion percentages between complete and partial adherent tumors (p = 0.005), and complete and non-adherent tumors (p<0.001). The improved technique demonstrated superior discriminatory ability in identifying tumor adhesion patterns compared to the previously described algorithm, with an AUC of 0.86 vs. 0.72 for distinguishing complete adhesion from others (p = 0.037), and an AUC of 0.72 vs. 0.67 for non-adherent and others. Aggressive tumors exhibiting atypical features showed significantly higher adhesion percentages in recurrence group compared to non-recurrence group (p = 0.042). This study validates the efficacy of the improved SII technique in quantifying meningioma adhesions and demonstrates its potential to affect clinical decision-making. The reliability of the technique, coupled with potential to help predict meningioma recurrence, particularly in aggressive tumor subsets, highlights its promise in guiding treatment strategies.

## Introduction

Meningiomas are the most common primary benign intracranial tumor, arising from the arachnoid cap cells. According to the WHO classification, meningiomas are histologically classified into three grades (I, II, and III) [1]. Approximately 80–85% of meningiomas are WHO grade I with benign features, 15–20% of meningiomas are WHO grade II with atypical features, and WHO grade III (anaplastic/malignant) make up 1–3% [2]. Although meningiomas are typically benign, the progressive enlargement of the tumors can compress adjacent neural tissue and result in neurological dysfunction or even life-threatening complications [3].

Surgery is a common therapeutic approach, yet the recurrence rates for meningiomas, particularly WHO grade II and III, remain high even after definitive treatment [4–7]. The recurrence rates for WHO grade II tumors range from 29% to 59%, while WHO grade III tumors show recurrence rates between 60% to 94% [8]. Nearly 95% of recurrent meningiomas grow in the same location and are of the same or higher tumor grade than the original tumor. Surgery combined with adjuvant radiotherapy was found an effective strategy after gross total resection in WHO grade II atypical meningiomas, it potentially decreases local recurrence [9–11]. Moreover, some researchers have identified a subset of WHO grade I that exhibits more aggressive atypical characteristics and significantly increased risk of tumor recurrence [8]. The impact of these atypical features on the risk of meningioma recurrence is not yet fully understood, and optimal treatment strategies remain uncertain. Identifying factors preoperatively that affect postoperative complications could assist in guiding clinical decisions, assessing treatment efficacy, and enhancing the overall surgical success rate of meningioma management [12].

Clinical experience has shown that the presence of adhesions between meningiomas and adjacent vital normal tissues, especially arteries and perforators, can significantly affect the extent of tumor resection and the likelihood of subsequent postoperative complications [13–15]. Adhesions lead to increased surgical intricacy, prolonged operative duration, extended recovery periods, and an elevated risk of neurological deficits [12]. This is especially critical as surgery and radiation have symbiotic effects on tumor control: surgery will reduce the overall treatment volume of radiation to reduce its risks, and radiation can treat adherent areas that, if removed, may result in permanent morbidity for the patient. In the context of predicting surgical outcomes and tumor recurrence in meningiomas, MRI emerges as a potent diagnostic tool, providing insights from tumor morphology, pathology, metabolism, and molecular characteristics [16–21]. While preoperative MRI has identified key prognostic markers like tumor size, location, shape, and adjacent bone invasion, the intricate interplay between tumor adhesiveness and meningioma recurrence remains largely elusive to conventional imaging techniques.

Ultrasound studies have investigated the integration of ultrasound elastography and MRI for brain tumor assessment, alongside advancements in intraoperative techniques [22, 23]. Moreover, machine learning classifiers have been employed to characterize meningioma consistency by combining radiomic features from MRI and ultrasound elastography, with potential applications for intraoperative strain ultrasound elastography [24, 25]. However, the assessment of adhesion via ultrasound, while valuable, is primarily intra-operative and faces limitations including shallow penetration depth, susceptibility to air or gas disruption, operator dependency, and image quality issues, which may impact reliability and interpretation [22, 23, 26, 27]. Preoperative assessment of tumor adhesion has employed various imaging modalities including CT and MRI [28, 29]. While digital subtraction angiography (DSA) shows promise for evaluating tumor adhesion, its invasive nature poses limitations [30]. Brain surface motion imaging, an MRI-based method, has developed as a noninvasive technique for predicting tumor-brain adhesion without contrast agents; however, variations are observed in the amplitude of cardiac cycle–dependent motion in MRI-detectable cerebrospinal fluid (CSF) flow across different intracranial locations, with minimal flow detected toward the vertex, which constrains its utility [31]. Furthermore, standard MRI may encounter challenges in visualizing specific tumor characteristics, potentially compromising accurate assessment of tumor adhesion [32].

MR elastography (MRE) [33] is an MR-based imaging technique capable of quantitatively measuring the mechanical properties of biological tissues in tumor assessment. With meningiomas, previous studies have demonstrated using MRE-derived information to sensitively assess the presence of adhesion between tumors and surrounding tissues. This is achieved through the evaluation of tumor movement relative to the brain parenchyma under the MRE-induced external dynamic vibrations. This MRE-based slip interface imaging (SII) technique directly assesses the relative motion between the tumor and surrounding brain parenchyma by measuring normalized octahedral shear strain (NOSS) [34, 35]. While qualitative evaluations of tumor adhesion have shown strong correlation with surgical outcomes, quantification of tumor adhesion remains a significant challenge [34]. Previous studies have quantified tumor adhesion by analyzing the NOSS characteristics across the tumor boundary (entropy of ΔNOSS_bdy_), using normal lines to that boundary as a reference [34]. However, our experience has shown that the accuracy of this approach can be affected by irregular tumor shape.

In this study we developed and tested an improved algorithm for SII, based on the recognition of NOSS image patterns. This slip interface recognition (SIR) algorithm is designed to measure the percentage of tumor margin that is adherent, providing a quantitative assessment of tumor adhesion. We compared the improved SIR method to the previously developed adhesion metric (entropy of ΔNOSS_bdy_), as well as to conventional MRI evaluations that are based on the presence of a CSF cleft. Additionally, we investigated the potential predictive value of MRE-based tumor adhesion in tumor recurrence, specifically within the subset of patients exhibiting atypical features of meningiomas.

## Materials and methods

### Patients

This prospective study was approved by the Mayo Clinic Institutional Review Board (IRB) under ID: 12–006661, titled "Magnetic Resonance Elastography of Meningiomas". All patients provided written informed consent prior to participation. From October 2013 to October 2021, a total of 57 patients with presumed meningiomas, confirmed by pathology at the time of surgery, underwent pre-operative brain MRI/MRE. Of these, 5 patients were excluded from the analysis due to: small tumor size (n = 2, maximum tumor diameter < 2.5 cm), lost to 1-year clinical and radiological follow-up (n = 2), and MRE technical failure (n = 1, low wave amplitude). Ultimately, 52 patients with meningiomas were included in the study for the following analysis. Some of the patient participants (45 of 52) are a subset from our previously published work [34].

### Pre-operative MRI/MRE acquisition

All patients were scanned on 3.0 T GE MRI scanners. MRE/SII data were acquired using a single-shot, flow-compensated, spin-echo (SE), echo-planner-imaging (EPI) MRE pulse sequence with a standard 8-channel receive-only head coil [35]. For MRE, low-amplitude mechanical vibrations at 60 Hz were introduced into the brain using a soft, pillow-like passive driver placed underneath the subject’s head, which was connected to an active driver (Resoundant, Inc., Rochester, MN). The resulting three-dimensional motion of the brain was encoded into the phase of the MR signal using the following imaging parameters: TR = 3600–4000 ms, TE = 58.7–64.3 ms, field of view (FOV) = 24 cm, acquisition matrix = 80 × 80 then reconstructed to 128 × 128 for NOSS processing, 48 continuous axial slices with a thickness of 3 mm, 2xASSET acceleration, 8 phase offsets sampled over one period of the 60 Hz motion, and 6 MRE motion encoding directions with ±*x*, ±*y*, and ±*z*. Additionally, a 3D T1-weighted (T1W) image was acquired using either an inversion recovery-prepared spoiled gradient echo (IR-SPGR) pulse sequence or a magnetization-prepared rapid gradient echo (MP-RAGE) pulse sequence. The T1W image was obtained with a sagittal orientation with a superior-inferior frequency-encoding direction. The specifications for the IR-SPGR were: TR/TE = 7.0/2.8 ms, flip angle = 11°, inversion time = 400 ms, FOV = 27 cm, acquisition matrix = 256 × 256, 196 slices with a spacing of 1.2 mm, and 1.75x array spatial sensitivity encoding technique acceleration. The MP-RAGE included: TR/TE = 7.4/3.0 ms, flip angle = 8°, inversion time = 900 ms, FOV = 26 cm; acquisition matrix = 256 × 256, and 166 slices with a spacing of 1.2 mm. Other MR imaging protocols included axial T2-weighted fast spin-echo, fast imaging employing steady-state acquisition (FIESTA), and T2-weighted fluid-attenuated inversion recovery (FLAIR), with the following acquisition parameters: T2-weighted fast spin echo (TR/TE = 3000–6000/102 ms, echo train length = 12, acquisition matrix = 256 × 256, FOV = 22 cm, and slice thickness = 4 mm), FIESTA (TR/TE = 7.6 ms/minimum full echo time, flip angle = 50°, acquisition matrix = 256 × 256, FOV = 12 cm, slice thickness, 1 mm), T2-weighted FLAIR (TR/TE = 11000/147 ms, inversion time = 2250 ms, acquisition matrix = 256 × 192, FOV = 22 cm, and slice thickness = 4 mm).

### Surgical grading of meningioma-brain adhesion

The surgical assessment of tumor adhesion to the adjacent brain tissue was conducted by an experienced neurosurgeon (J.J.V.G., with 17 years of experience) in a blinded manner to the SII results, following the methodology previously described [35]. The degree of tumor adhesion was categorized into three groups: complete adhesion, partial adhesion, and no adhesion, with specific definitions as follows:

- Complete adhesion: In cases where the tumor could not be easily separated from the adjacent brain tissue, dissection had to be performed subpially in more than 2/3 of the total tumor-cortex interface.
- Partial adhesion: For instances where a clear surgical plane was partially lost, and the pial membrane was adherent to the tumor in more than 1/3 but less than 2/3 of the interface.
- No adhesion: In situations where a clear surgical plane was identified in more than 2/3 of the surface between the tumor capsule and the brain surface, indicating absence of adhesion.

### MR Imaging-based of tumor-brain CSF cleft

A neuroradiologist (J.H., with 34 years of neuroradiology experience) assessed the ability of standard MR imaging to predict tumor adherence, blinded to surgical outcomes. A peritumoral CSF cleft was defined as a thin layer with high signal intensity on T2-weighted and/or FIESTA images and low signal intensity on T2-weighted FLAIR images at the tumor-brain interfaces [36]. The grading of CSF clefts was based on their extent across the tumor surface and classified into three categories:

- Complete cleft: A cleft is present, and it involves greater than 2/3 of the tumor-brain interface, suggesting no adhesion.
- Partial cleft: A cleft is present and involves between 1/3 and 2/3 of the tumor-brain interface, suggesting partial adhesion.
- No cleft: No evidence of a CSF cleft between the tumor and normal brain, or presence of a cleft less than 1/3 the tumor-brain interface, suggesting complete adhesion.

### Tumor ROI segmentation

The 3D manual segmentation of these tumors was conducted by defining the boundaries of the tumors on each individual slice, covering the entire tumor volume, by a neurological research fellow (E.C., with 2 years of imaging research) supervised by an experienced neuroradiologist (J.H., with 34 years of expertise). Brain masks were generated by segmenting the T1W images and calculating probabilistic maps of gray matter, white matter, and CSF with the use of SPM5 [35]. A brain mask was created by considering voxels in which the combined gray and white matter content exceeded the CSF content. The T1W image, brain mask, and tumor mask were then registered and resampled to match the MRE space. Trilinear interpolation was used for resampling the T1W image while the brain and tumor masks were resampled using nearest-neighbor interpolation. Of these 52 patients, 45 patients were previously published with a separate tumor ROI delineated in a prior publication [34]. We specifically used this subset of tumors with two tumor ROIs to conduct an Intraclass Correlation Coefficient (ICC) analysis to investigate the dependency of our algorithm on tumor segmentation.

### Tumor adhesion assessments by slip interface recognition (SIR)

**Fig 1:**
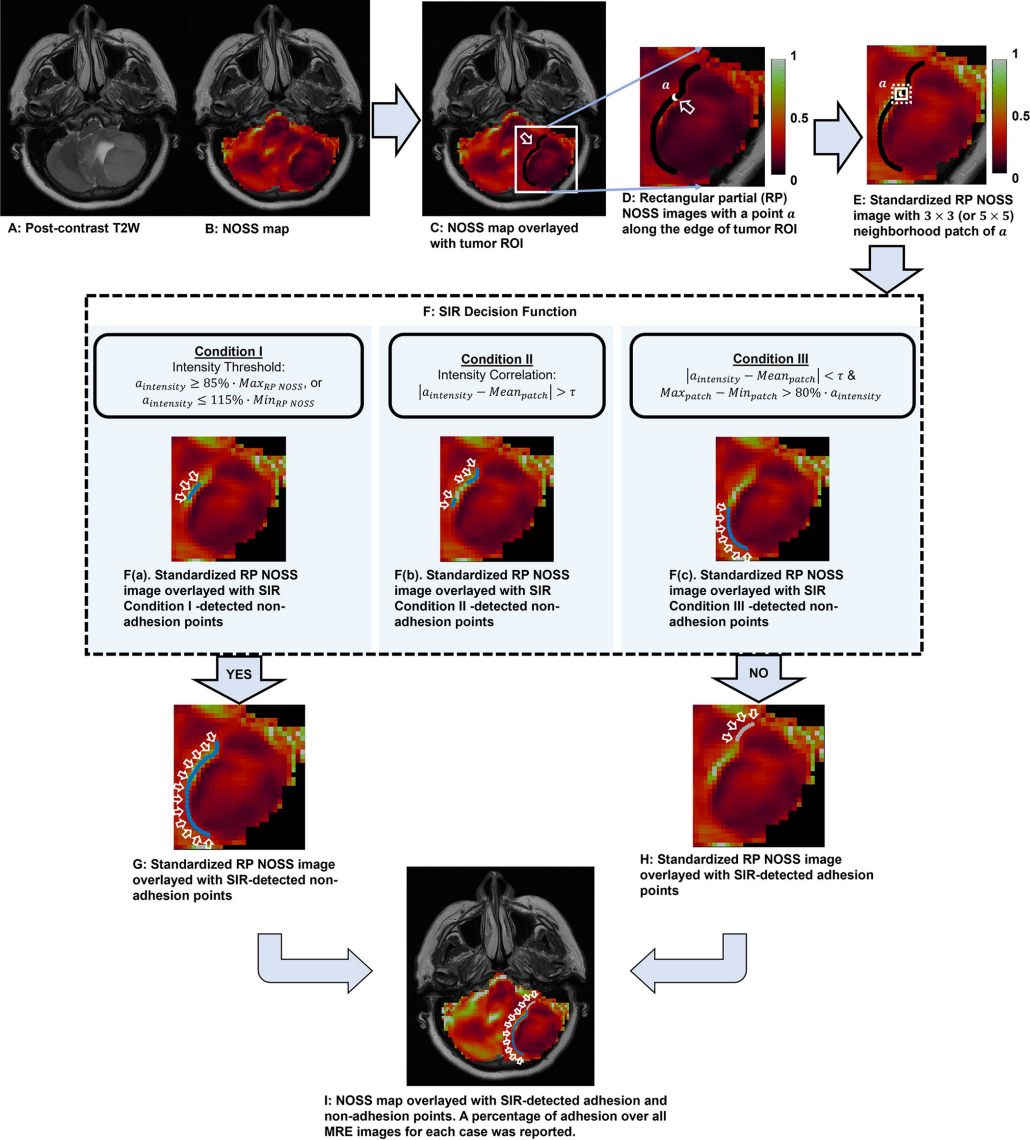
Flowchart illustrating the process of slip interface recognition (SIR) for a case of left posterior fossa meningioma (62-years-old, female), which is non-adhesion of surgical adhesion grading. (A) T2-weighted image of the tumor. (B) The corresponding NOSS map. (C) Tumor ROI overlaid on the NOSS map, indicated by a black line in the white rectangle. (D) Extraction of rectangular partial (RP) NOSS images by extending 3 pixels (or 5 pixels) in the horizontal and vertical directions beyond the tumor ROI boundary. (E) Standardization of the intensity of RP NOSS images. For each point *a* along the edge of the tumor ROI, a neighborhood patch was identified with a default size of 3×3 (white square) or increased to 5×5 (white dashed square). (F) Non-adhesion at each point was determined by the SIR Decision Function with a value of “Yes” if any of the three conditions were met. Non-adhesion points determined by the SIR decision function Condition I, II, or III exclusively, were represented in F(a), F(b), and F(c), respectively. The non-adhesion points were depicted the blue line on the standardized RP NOSS image, indicated by white arrows. (G) All non-adhesion points (blue line) and (H) adhesion points (grey line) determined through the combination of points identified by the SIR decision function under Conditions I, II, and III. (I) SIR automatically classifies the non-adherent (blue line) and adherent (light gray line) interfaces and calculates the non-adhesion and adhesion percentage over the 3D tumor surface (37.71% of adhesion percentage in this case).

We computed the NOSS maps based on the measured displacement fields obtained from the unwrapped phase image for each slice of the MRE images of each patient, following the method described in [35]. The octahedral shear strain (OSS) was calculated for each phase offset and averaged over the eight offsets [37]. The NOSS map (**Fig 1B**) was calculated by normalizing the OSS with a combined amplitude calculated as the square root of the sum of squares of the first harmonic of the complex shear waves along the *x*/*y*/*z*-axis, where *x*, *y*, and *z* represent the left-to-right, anterior-to-posterior, and superior-to-inferior directions in MRI scans, respectively.

The presence of a hyper-intense NOSS tumor interface is typically observed at slip boundaries, where the tumor lacks adherence to the adjacent brain parenchyma. Conversely, a lack of a high-intensity NOSS contrast along the tumor interface usually indicates adhesion to surrounding tissue [34]. To objectively identify adherent vs. non-adherent boundaries, we developed and tested an improved algorithm termed “slip interface recognition” (SIR), that leverages recognition of patterns in NOSS images to automatically identify tumor adhesion by analyzing the given tumor boundary and the characteristics of its neighborhood. Because the tumor ROI was delineated manually, it may be slightly misaligned with the actual tumor margins. In such cases, the presence of hyper-intense NOSS contrast may result in high-intensity features appearing 1–2 pixels away from the delineated tumor margin. The manually delineated boundary would lie in regions adjacent to the high-intensity features, which display either distinct low-intensity features or a significant intensity variation. In this context, the SIR categorized these distinct low-intensity features and significant intensity variations as non-adhesion. Moreover, due to variations in image quality, subtle details in the NOSS image may be difficult to discern by visual inspection, and discrepancies between observers could lead to inaccurate subjective observations. Using surgical adhesion grading as the gold standard, we observed that tumors with a large variation in intensity along the tumor interface in the NOSS image, which may not be discernible to visual observers due to image quality, can also indicate non-adhesion. A case for illustration is shown in S1 Fig.

#### NOSS image pre-processing

Due to the complexity of intracranial changes caused by tumor compression, and the characteristics of NOSS images at the tumor boundary, the SIR analysis utilizes the intensity of points along the tumor margin and within the adjacent 3x3 or 5x5 neighborhoods patches on the NOSS image to study the characteristics of tumor adhesion and tumor non-adhesion. Before proceeding with further analysis, rectangular portions (RPs) of the NOSS images were extracted by extending three pixels beyond the largest extent boundaries of the given tumor ROI in both horizontal and vertical directions, as shown in **Fig 1D**. To ensure consistent contrast across all patients, the maximum intensity of the RP NOSS images was empirically standardized to 1, as illustrated in **Fig 1E**.

#### Feature extraction and data training

A subset of the data (15%) obtained from the 52 patients was used to train the SIR algorithm. Surgical evaluations of tumor adhesion served as the gold standard, where meningiomas were classified into three groups as defined above: completely adherent, partially adherent, and non-adherent [34].

Next, neighborhood patches were identified by tracking the edge points of the tumor ROI with a default size of 3 × 3. When the 3 × 3 patch did not include sufficient extracted tumor boundary information, the patch size was increased to 5 × 5. Specifically, if the difference between the average intensity of a 3 × 3 patch (comprising 9 pixels) and the intensity of a point *a* along the edge of the given tumor ROI was less than an empirically derived threshold of 0.065 (based on the training dataset), we increase the patch size to 5x5. This adjustment allows for a more comprehensive extraction of tumor boundary information.

Based on the pattern features along the tumor margin in the NOSS images, where hyper-intense NOSS, distinct low-intensity and large intensity variations in NOSS image along the tumor interface indicates a non-adhesive tumor, we established three conditions associated with tumor non-adhesion. For each point, non-adhesion (Yes) was determined if any of 3 conditions were satisfied:

Condition I: The intensity of the point *a* fell outside 85% of the maximum intensity and 115% of the minimum intensity of the RP NOSS images (**Fig 1F(a)**). This condition corresponds to a very clear NOSS boundary that is easily identifiable by any observer. Such boundaries exhibited distinct high-intensity along tumor boundary, or distinct low-intensity features when the tumor ROI and actual tumor margin were not perfectly aligned.

Condition II: The difference between the intensity of point *a* and the average intensity of the corresponding neighborhood patch was greater than a threshold *τ*, which was empirically set as 20% of the difference between the maximum and minimum intensities of the patch (**Fig 1F(b)**). This condition represents a less distinct intensity contrast at the tumor boundary compared to Condition I, yet it remains discernible and can be traced along the margin. This condition also included situations featuring significant intensity variations in NOSS images when the tumor ROI and actual tumor margin were misaligned.

Or condition III: When the difference between point *a* intensity and the average intensity of the corresponding neighborhood patch by less than the threshold *τ*, the difference between the maximum and minimum intensity of the patch is greater than 80% of the intensity of the point *a* (**Fig 1F(c)**). This condition corresponds to interfaces that display large intensity variations in the neighboring patches along the tumor margins, interfering with the visual inspection and presenting tumor boundaries that show relatively weak or faint contrast with the surrounding brain tissue.

The non-adhesion percentage was calculated for all tumor slices for each patient. The output measures the percentage (%) of the peritumoral interface length with shear strain shown to be non-adhesion across all tumor-brain interfaces, as indicated by the blue arrow in **Fig 1G**. **Fig 1I** displays a visual representation of adhesion percentage on the NOSS image. For comparison, another adhesion metric, the entropy of ΔNOSS_bdy_, was calculated as previously described [34]. The discriminative capabilities of both the SIR-derived adhesion percentage and the entropy of ΔNOSS_bdy_ were then evaluated on all 52 tumors based on surgical adhesion grading.

### Clinical data collection and tumor recurrence follow-up

Tumors were classified as skull base and non-skull base in location. Skull base tumors were defined by the following locations: cavernous sinus, clinoidal, cerebellopontine angle, foramen magnum, anterior fossa, clival, and petroclival. All other tumors were classified as non-skull base.

Recurrence was defined as new nodular enhancement in the resection cavity in the gross total resection or enlargement of a residual tumor by MRI and confirmed by clinical review. The tumor tissue was graded using the WHO 2016 CNS tumors criteria [38]. Atypical features were significantly associated with the recurrence rate of meningioma, including Grade I tumors with atypical features [8]. Therefore, the presence of atypical features was recorded for both Grade I and Grade II meningiomas, and patients were grouped into those with atypical features (including atypical WHO Grade II and Grade I with atypical features) and those with non-atypical features. Atypical features were defined as the presence of increased cellularity, sheeting, prominent nucleoli, high nuclear-to-cytoplasmic ratio, or presence of necrosis.

### Statistical analysis

The statistical analysis was conducted using BlueSky Statistics software (v10, BlueSky Statistics LLC, Chicago, IL, USA). Continuous variables were reported as mean values with standard deviation, while categorical variables were presented as frequencies and/or percentages. For group comparisons among different surgical adhesion grades, nominal data were analyzed using the Chi-square test (or Fisher exact test when appropriate). Wilcoxon rank-sum test and one-way ANOVA were used for continuous variables when appropriate. A post-hoc Turkey honestly significant difference (HSD) test was performed for the ANOVA. ICC analysis was used to assess the dependency of our SIR algorithm on tumor segmentation. An ICC between 0.75 and 0.9 reflects good reliability of the algorithm, while values exceeding 0.9 indicate excellent reliability. The agreement between CSF cleft predictions and surgical findings was assessed using Cohen’s κ coefficients, with interpretations categorized as poor (<0.20), fair (0.21–0.40), moderate (0.41–0.60), or good (>0.60) agreement [39]. To investigate the correlation with tumor recurrence, univariate analysis was performed first, then multivariate regression analysis was conducted, which included characteristic parameters showing a p-value < 0.1 at the univariate regression analysis. In addition, to compare the classification efficacy of SIR with the previous technique (entropy of ΔNOSS_bdy_), receiver operating characteristic (ROC) analysis was performed by calculating the area under the curve (AUC) values and diagnostic accuracy for discerning the extent of adhesion. DeLong’s test was employed to compare the two distinct ROCs, and the AUC value was calculated. A p-value of less than 0.05 was considered statistically significant.

## Results

### Demographics and clinical presentation

**Table 1** summarizes the demographic and clinical characteristics of 52 patients. The mean age of the patient population in this study was 59.9 ± 11.2 years (range 30–81 years), with the majority being female (73.1%, 38 out of 52). The most prevalent tumor location was the skull base (27 patients, 51.9%), and the mean tumor size was 4.6 ± 1.4 cm (range 2.5–9 cm). The majority of the tumors were WHO grade I (80.8%, 42 out of 52) and 16 patients (30.8%, 16 out of 52) presented with atypical features. Tumor recurrence was found in 10 out of 52 (19.2%) patients. The mean adhesion percentage was measured as 43.6% ± 10.1% of patients (range 20–67.2%). Complete adhesions were observed in 11 cases (21.2%), partial adhesions in 19 cases (36.5%), and no adhesions in 22 cases (42.3%). Upon analysis of the three subgroups of surgical adhesion grading, no significant correlations were observed with tumor adhesion concerning age, sex, tumor size, tumor location, and tumor recurrence. A noteworthy trend in the correlation between SIR-measured adhesion percentage (p < 0.001) and WHO Grade had a marginally significant p-value of 0.068.

**Table 1:** Demographic data and clinical characteristics of patients with meningioma (N = 52).

Characteristic	All patients (N = 52)	Complete Adhesion (N = 11)	Partial Adhesion (N = 19)	Non-Adhesion (N = 22)	p-value
Age (years)	59.9 ± 11.2 (Range [30, 81])	62.7 ± 10.1 (Range [46, 81])	59.1 ± 11.9 (Range [30, 80])	59.3 ± 10.9 (Range [38, 80])	0.66
Sex (Male/Female)	14/38	5/6	5/14	4/18	0.25
Tumor Size (cm)	4.6 ± 1.4 (Range [2.5, 9])	4.8 ± 1.1 (Range [3, 7])	4.7 ± 1.5 (Range [2.8, 9])	4.5 ± 1.3 (Range [2.5, 6.7])	0.88
Tumor Location (Skull base/non-Skull base)	27/25	7/4	11/8	9/13	0.38
WHO Grade (Graded I/Grade II)	42/10	8/3	13/6	21/1	0.068
Features (Non-Atypical/Atypical)	36/16	6/5	13/6	17/5	0.41
Adhesion (%)	43.64 ± 10.10 (Range [20.91, 63.16])	52.93 ± 7.16 (Range [41.9, 63.16])	42.18 ± 6.90 (Range [28.06, 51.33])	37.89 ± 9.93 (Range [20.91, 56.13])	<0.001
Recurrence (Recurrence /Non- Recurrence)	10/42	4/7	4/15	2/20	0.17

Within the cohort of 42 patients initially classified as WHO Grade I, subsequent analysis identified the presence of atypical features in 6 of these 42 patients. As a result, with the addition of 10 patients with WHO II atypical features, the cohort was reclassified into 36 patients with non-atypical features, and 16 patients with atypical features.

### Correlation between SIR adhesion estimation and surgical adhesion grading

**Fig 2:**
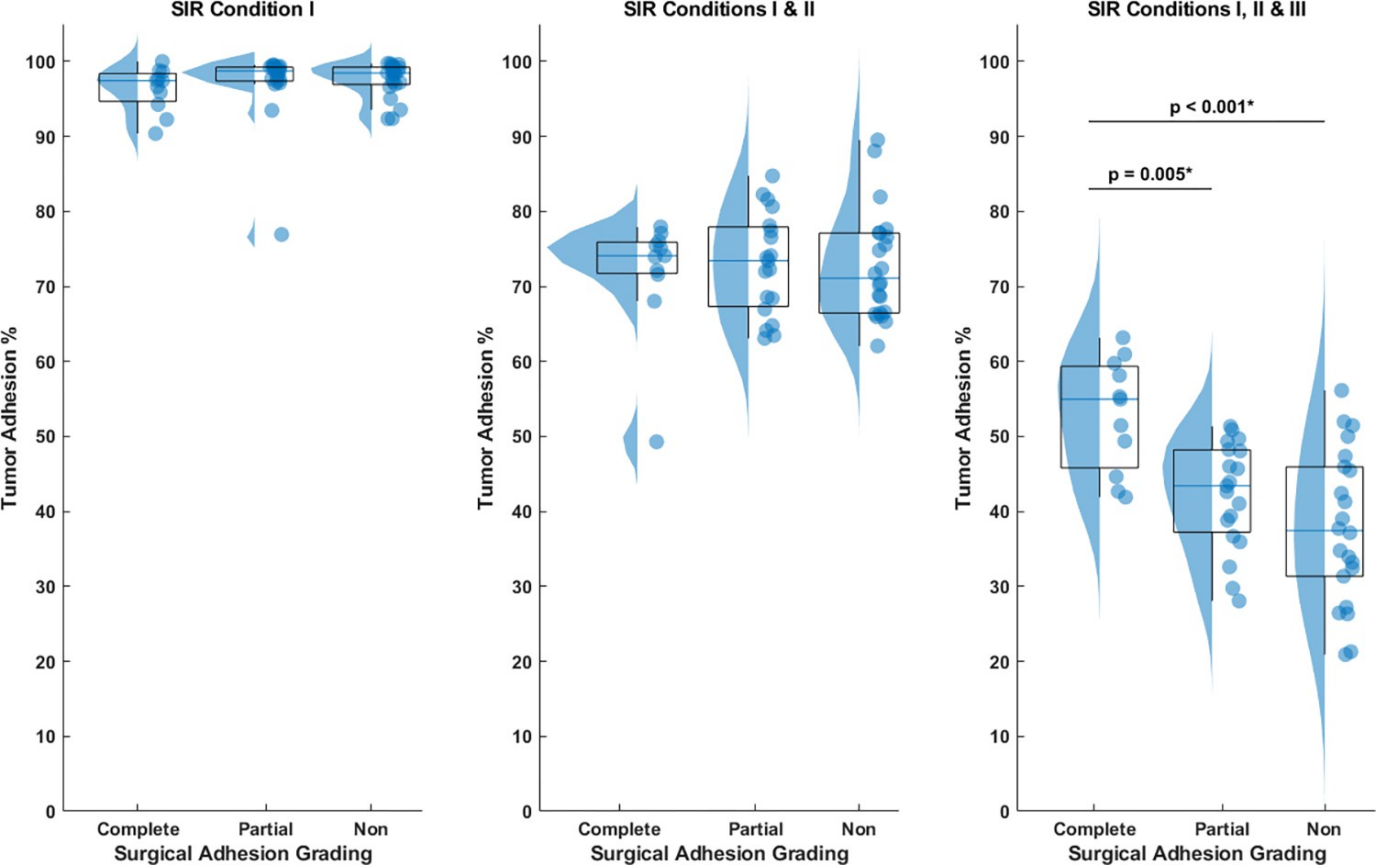
Raincloud plots comparing the tumor adhesion percentage based on surgical adhesion grades when using different SIR conditions. (a) Application of solely SIR condition I, or (b) both conditions I and II failed to differentiate among the three tumor categories based on tumor adhesion percentage. (c) Employing all three SIR conditions reveals that complete adhesion tumors exhibit a higher tumor adhesion percentage compared to partial and non-adherent tumors.

To illustrate the importance of employing all three SIR conditions, **Fig 2** presents raincloud plots that compare tumor adhesion percentages based on surgical adhesion grades when using just Condition I, both Conditions I and II, and all three Conditions, respectively. In **Fig 2(A) and 2(B)**, utilizing only SIR condition I, or conditions I and II, do not yield clear differentiation among the three tumor categories based on tumor adhesion percentage. Advancing further, **Fig 2(C)** incorporates all SIR conditions I, II, and III—revealing that tumors with complete adhesion display a significantly higher tumor adhesion percentage compared to both partial and non-adherent counterparts (p = 0.005 and p < 0.001, respectively). Moreover, a discernible trend in adhesion percentage emerges between partial and non-adherent tumors, with partially adherent tumors exhibiting a noticeably higher median adhesion percentage relative to non-adherent tumors.

Forty-five among the 52 meningioma patients in our study had been published with separate delineations of ROIs [34]. We conducted an ICC analysis within this sub-cohort of patients to assess the dependence of our improved technique on tumor ROI delineation. We found an inter-observer ICC of 0.89, demonstrating the reliability of our approach.

### Comparing SIR-derived adhesion percentage to the entropy of ΔNOSS_bdy_ and peritumoral CSF cleft

**Fig 3:**
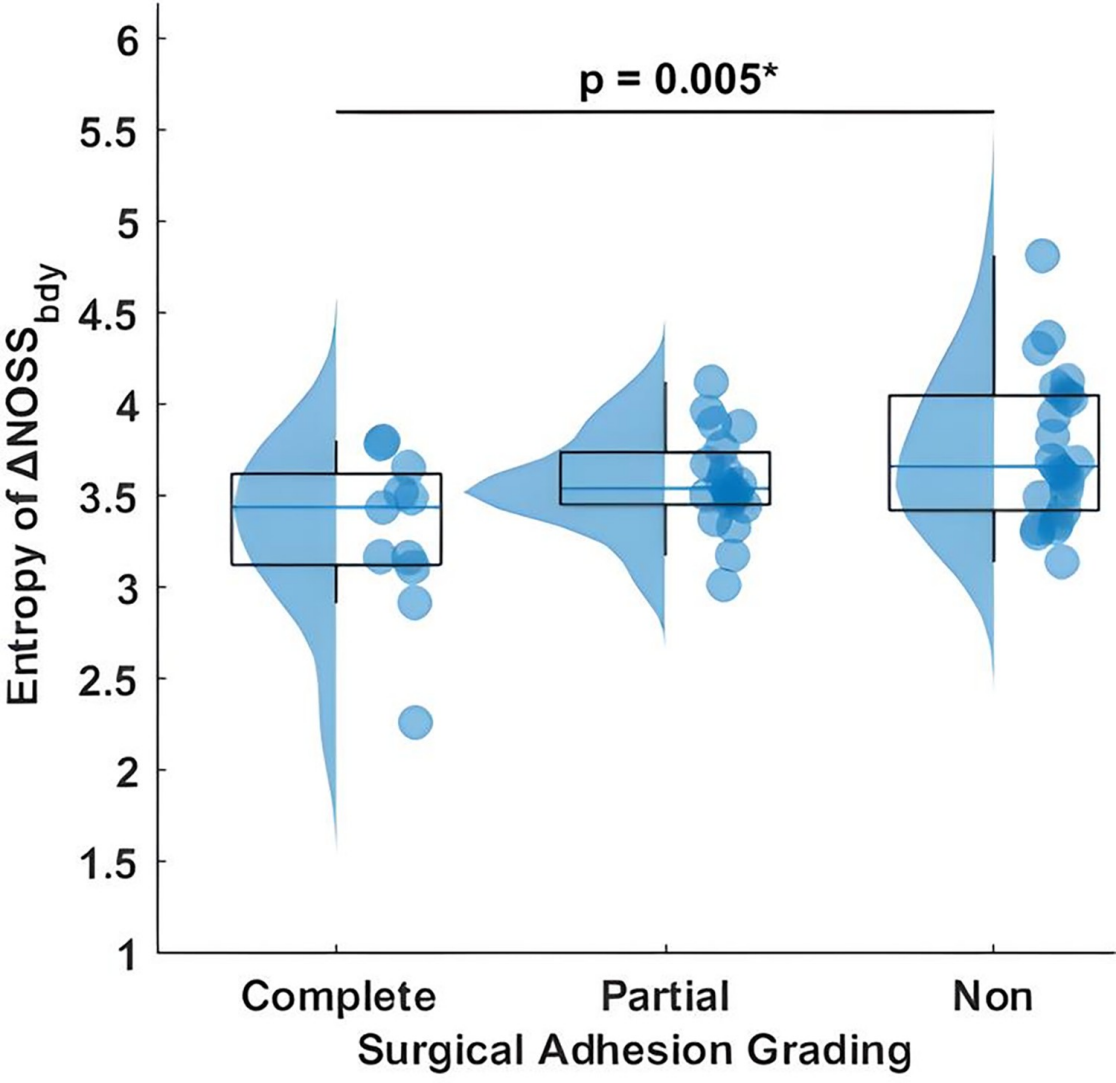
Raincloud plot of entropy of ΔNOSS_bdy_ relative to surgical adhesion grading, encompassing complete, partial, and non-adherent tumor categories. A significant distinction is observed between complete and non-adherent tumors.

**Fig 4:**
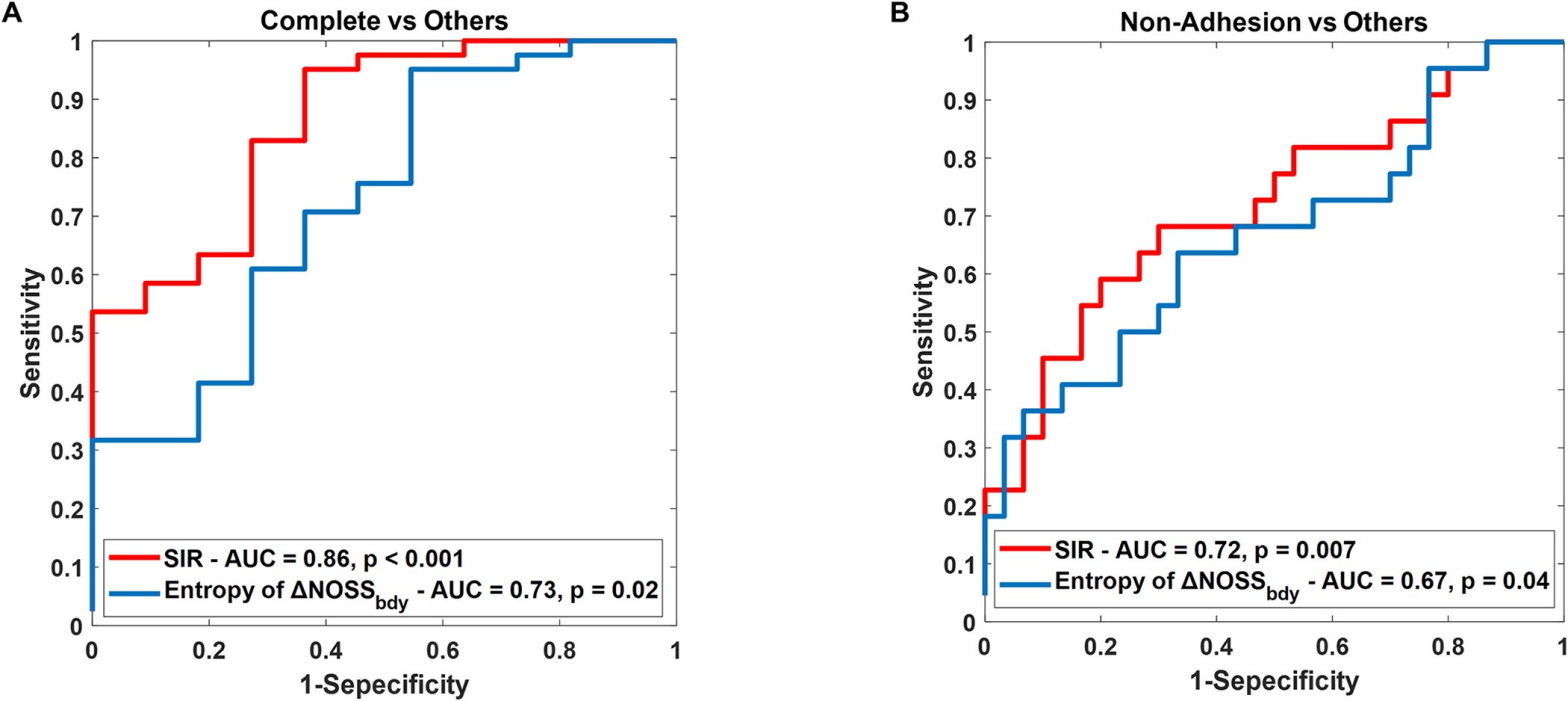
The diagnostic performance of two algorithms, SIR and entropy of ΔNOSS_bdy_, within a cohort of meningioma patients (N = 52). ROC curves for (a) complete adhesion versus partial/no adhesion, and (b) non-adhesion versus partial/complete adhesion.

We compared the diagnostic efficacy of SIR-derived tumor adhesion percentage and the previously reported entropy of ΔNOSS_bdy_ for the identification of tumor adhesion among meningioma patients. **Fig 3** shows a raincloud plot of entropy of ΔNOSS_bdy_ with surgical adhesion grading. A significant difference is observed between complete and non-adherent tumors (p = 0.005). **Fig 4** and **Table 2** compares the diagnostic performance of (a) complete adhesion versus partial/no adhesion and (b) non-adhesion versus partial/complete adhesion.

**Table 2:** The area under the curve (AUC) values and p-values for SIR and entropy of ΔNOSS_bdy_ and DeLong’s test compared to assess the discriminative power of SIR and entropy of ΔNOSS_bdy_.

	SIR	Entropy of ΔNOSS_bdy_	DeLong’s Test
			p-value
Complete vs Others	0.86	0.73	0.037*
	(p < 0.001)	(p = 0.02)	
Non-Adhesion vs Others	0.72	0.67	0.4
	(p = 0.007)	(p = 0.04)	

In the comparison of complete adhesion versus partial/no adhesion, the AUC for SIR was 0.86 and for the entropy of ΔNOSS_bdy_ was 0.72. The difference in AUCs between the two techniques was statistically significant (p = 0.037) according to DeLong’s test. SIR thus demonstrates superior classification performance in distinguishing complete adherent tumors from others. In the comparison of non-adhesion versus partial/complete adhesion, the AUC for SIR was 0.72 and for entropy of ΔNOSS_bdy_ was 0.67; however, the difference was not statistically significant (p = 0.4).

Additionally, we assessed our SIR-derived measurements with standard MRI evaluations by examining peritumoral CSF cleft. **Table 3** demonstrates a *κ* coefficient of 0.057 [95% CI: -0.13, 0.24] indicating poor agreement between CSF cleft predictions and surgical findings. The *κ* coefficient is not applicable for assessing the correlation between the SIR and surgical findings, as the SIR-derived adhesion is quantified. However, visual representations in **Figs 2 and 4**, complemented by data in **Table 2**, provide insights into the alignment between SIR-derived measurements and surgical outcomes.

**Table 3:** Case agreement and *κ* coefficient for agreement between peritumoral CSF cleft and surgical findings.

	Surgical Fundings			*κ* Coefficient (95% CI range)
	Complete Adhesion	Partial Adhesion	Non-Adhesion	
CFS Cleft				0.057 (-0.13, 0.24)
Complete Cleft	4	7	6	
Partial Cleft	5	9	10	
Non-Cleft	2	3	6	

### Variables associated with recurrence

**Fig 5:**
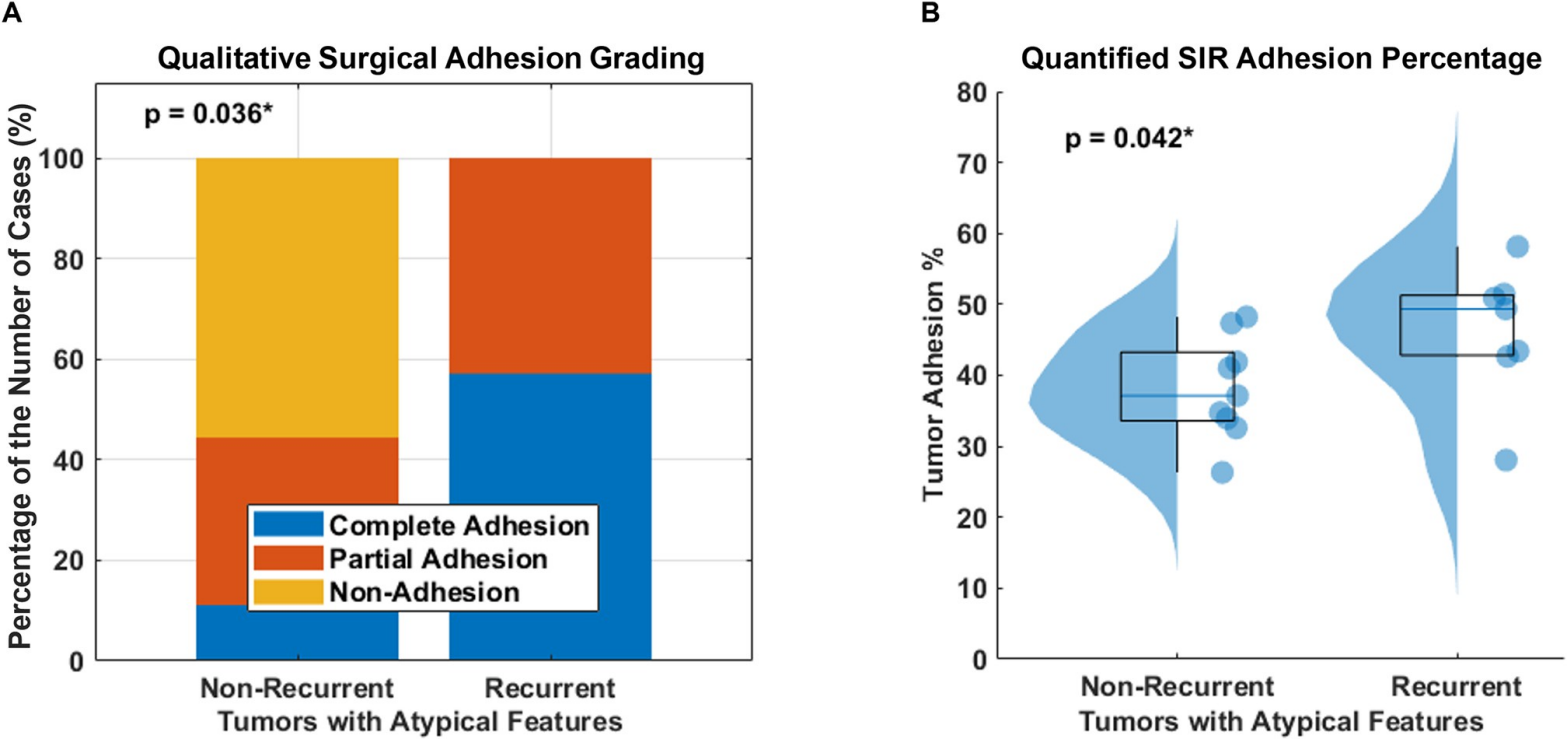
Group comparison between non-recurrent and recurrent tumors with atypical features. (a) Stacked bar charts show the percentage distribution of the number of each qualitative surgical adhesion grading with atypical features, and (b) SIR-derived tumor adhesion percentage with atypical features.

**Table 4** provides a summary of the relationship between various pre-operative and post-operative clinical variables and tumor recurrence. In the overall patient cohort, significant differences were found between the recurrence and non-recurrence groups for the WHO grading (p = 0.0016) and the presence of atypical features (p = 0.0057), but not in the qualitative adhesion surgical grading (p = 0.17) and SIR-derived adhesion percentage (p = 0.42). Further investigation revealed that among all aggressive tumors with atypical features, we found that tumor adhesion was significantly associated with tumor recurrence and demonstrated that qualitative surgical adhesion grading was correlated with recurrence (p = 0.036), and SIR quantitatively showed that the adhesion percentage in the recurrence group was significantly higher than that in the non-recurrence group (p = 0.042), as demonstrated in **Fig 5**.

**Table 4:** Demographic data and clinical characteristics of meningiomas with and without recurrence.

	Recurrence	Non-Recurrence	Univariate Analysis	Multivariate Analysis
			p-value	p-value
Number	10 (19.2%)	42 (80.8%)		
Age (years)	61.2 ± 18.1 [30, 81]	59.6 ± 9.4 [39, 80]	0.62	
Sex			0.11	
Male	5 (50%)	9 (21.4%)		
Female	5 (50%)	33 (78.6%)		
Tumor Size (cm)	4.4 ± 1.2 [2.5, 6.2]	4.7 ± 1.4 [2.5, 9]	0.55	
Tumor Location			1	
Skull base	5 (50%)	22 (52.4%)		
Non-Skull base	5 (50%)	20 (47.6%)		
WHO Grade			0.0016**	0.0014**
Grade I	4 (40%)	38 (90.5%)		
Grade II	6 (60%)	4 (9.5%)		
Feature			0.0057**	
Atypical	7 (70%)	9 (21.4%)		
Non-Atypical	3 (30%)	33 (78.6%)		
Qualitative Adhesion Surgical Grading			0.17	
Complete	4 (40%)	7 (16.7%)		
Partial	4 (40%)	15 (35.7%)		
Non	2 (20%)	20 (47.6%)		
Quantified SIR Adhesion (%)	44.95 ± 7.84 [28.06, 58.14]	42.09 ± 10.49 [20.91, 63.16]	0.42	

## Discussion

In the neurosurgical community, it is widely acknowledged that meningioma adhesion has major impact on surgical and clinical outcomes. As a result, many studies have examined pre-operative MRI findings to predict tumor adhesion [40–43]. Previous studies have indirectly assessed tumor adhesive tendencies with MRI by measuring tumor vascular distribution and peritumoral cerebrospinal fluid dynamics [28, 44–46]. However, the assessments of tumor mechanical properties in these studies have been indirect and subjective, leading to considerable observer variability. In our study, we utilized MRE-based imaging parameters for a more standardized and objective characterization of tumor adhesion. To achieve this, we utilized MRE-based shear strain mapping and developed and tested an improved algorithm (SIR) based on pattern recognition of strain characteristics at the tumor boundary to estimate the degree of tumor adhesion. These tumor characteristics have the potential to improve our understanding of meningioma biomechanical properties, ultimately contributing to optimized treatment options for patients.

The SIR technique involves the identification of NOSS image patterns present at the tumor boundary and subsequently utilizing these patterns to predict tumor adhesion. For the subset of patients previously published (45 out of 52), two radiologists delineated ROIs independently, which yielded an ICC of 0.89 in adhesion percentage, indicating the reliability of our algorithm in accommodating variations in tumor ROI delineation. Based on 3x3 or 5x5 pixel patches extracted from the NOSS image along the tumor margin, SIR extracted relevant information from these pixels to discriminate tumor adhesion patterns by capturing variations in intensity within the NOSS image. SIR can identify patterns within data that might be imperceptible to the human eye, potentially contributing to consistent recognition while helping to mitigate interobserver errors and biases.

The incorporation of multiple SIR conditions demonstrated enhanced sensitivity in distinguishing complete adhesion tumors from partial and non-adherent tumors. Furthermore, the SIR technique did encounter a certain degree of complexity in distinguishing between partial adhesions and non-adhesions. This may be partly attributed the small sample size within our study, and partly influenced by the subjective nature of our reference standard, where surgeon assessments were based on clinical records and impressions. Further research with more patients will allow the SIR algorithm to evolve further and improve the accuracy of tumor adhesion recognition. Comparing our SIR methodology with the previously reported entropy of ΔNOSS_bdy_ [34], both approaches utilize NOSS images but diverge in their analytical approaches and diagnostic applications. SIR demonstrated improved diagnostic capabilities compared to entropy of ΔNOSS_bdy_ in distinguishing tumor adhesion patterns, particularly in complete adhesion. Our findings underscore the enhanced reliability of SIR compared to entropy of ΔNOSS_bdy_. Furthermore, the standard MRI evaluations by examining the CSF cleft showed suboptimal concordance with surgical findings, further suggesting the superior predictive capability of the SIR methodology compared to conventional assessments.

To the best of our knowledge, our study represents the first investigation into the relationship between mechanical properties measured by MRE and the surgical/clinical outcomes in patients undergoing meningioma resection. Consistent with previous studies [8, 47–49], there is a significant correlation between histological subtypes and tumor recurrence, with high-grade tumors or low-grade tumors with atypical features having a higher likelihood of recurrence. Meningiomas with atypical features, such as increased mitotic activity, or invasion of adjacent structures, are considered more aggressive and can lead to an increased recurrence rate and overall morbidity. Notably, we found that tumor adhesion had no significant impact on tumor recurrence in the overall patient cohort. However, when focusing on aggressive tumors specifically, tumor adhesion was found to play a significant role. Specifically, aggressive tumors exhibiting adherence to surrounding structures may increase the risk of recurrence more than those without adhesion. We hypothesize that tumor adhesion may reflect the extent of invasiveness, as a higher adhesion percentage may indicate pial or perineural invasion of the tumor into the surrounding structures, which would lead to a higher likelihood of not achieving a gross total resection, with a resulting higher recurrence rate. These findings emphasize the importance of considering tumor adhesion as a factor in predicting tumor recurrence.

It is important to acknowledge the limitations of our study. Firstly, accurate tumor segmentation is crucial for calculating the tumor adhesion percentage. Manual segmentation was performed in our study. Although SIR was designed to accommodate slight mismatches between tumor ROI and the actual tumor boundary, manual ROI drawing can still be a time-consuming and labor-intensive process. Future studies could develop machine learning algorithms for automatic NOSS image pattern analysis, eliminating the need for manual tumor boundary demarcation. Secondly, the resolution of our current SII analysis is not sufficient to distinguish the adhesion between a tumor and adjacent very small structures. Higher resolution SII may help address this in the future [50]. Finally, the sample size of our study was relatively small. Further research with larger sample sizes and more diverse patient populations is necessary to confirm the findings of this study and to better understand the impact of tumor adhesion on surgical and clinical outcomes.

## Conclusion

In conclusion, this study developed and tested an improved SII-based algorithm to measure tumor adhesion in patients with meningiomas. This improved approach not only facilitates quantification but also contributes to the precise classification of meningioma-brain adhesion. Our findings provide initial evidence of the potential of MRE-measured tumor adhesion as promising biomarkers to predict surgical and clinical outcomes among patients with meningiomas. This could lead to more effective and personalized care strategies for managing meningiomas, ultimately improving patient outcomes.

## Supporting information

S1 Fig This case shows a 53-year-old female patient diagnosed with meningioma. The surgical grading categorized the tumor as non-adhesive. In (A), the figure depicts the challenge of visually discerning tumor adhesion at the edge of the tumor, as indicated by the white arrow. Our SIR technique, shown in (B), successfully identifies the adhesion as non-adhesive, depicted by the yellow line.
(TIF)
