# Supplementary figures and images for: Improved quantification of tumor adhesion in meningiomas using MR elastography-based slip interface imaging
Source: PLoS One. 2024 Jun 25;19(6):e0305247. doi: 10.1371/journal.pone.0305247 (PMC11198761; doi:10.1371/journal.pone.0305247)

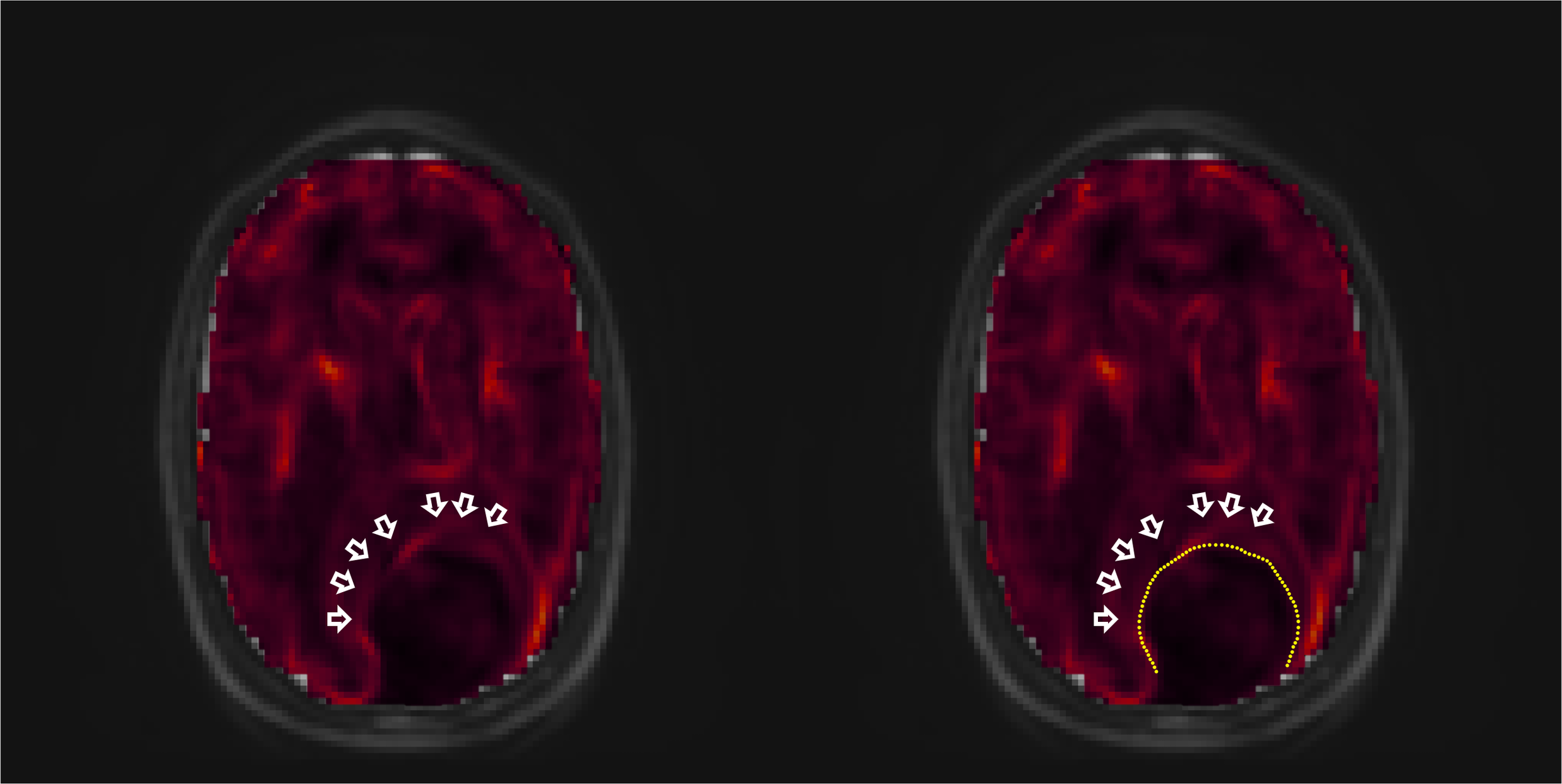

Supplement: S1 Fig — The surgical grading categorized the tumor as non-adhesive. In (A), the figure depicts the challenge of visually discerning tumor adhesion at the edge of the tumor, as indicated by the white arrow. Our SIR technique, shown in (B), successfully identifies the adhesion as non-adhesive, depicted by the yellow line. (TIF) [file pone.0305247.s001.tif]
